# Histone demethylases in the regulation of immunity and inflammation

**DOI:** 10.1038/s41420-023-01489-9

**Published:** 2023-06-23

**Authors:** Lihua Qu, Tong Yin, Yijin Zhao, Wenting Lv, Ziqi Liu, Chao Chen, Kejun Liu, Shigang Shan, Rui Zhou, Xiaoqing Li, Huifen Dong

**Affiliations:** 1 Hubei Province Key Laboratory of Allergy and Immunology, Wuhan University, Wuhan, Hubei China; 2 Department of Pathogenic Biology, School of Basic Medical Sciences, Wuhan University, Wuhan, Hubei China; 3 School of Basic Medical Sciences, Xianning Medical College, Hubei University of Science and Technology, Xianning, Hubei China; 4 School of Medicine & Holistic Integrative Medicine, Nanjing University of Chinese Medicine, Nanjing, Jiangsu China; 5 Biological Targeted Therapy Key Laboratory in Hubei, Huazhong University of Science and Technology, Wuhan, Hubei China; 6 Center for Stem Cell Research and Application, Union Hospital, Tongji Medical School, Huazhong University of Science and Technology, Wuhan, Hubei China

**Keywords:** Epigenetics in immune cells, Innate immune cells

## Abstract

Pathogens or danger signals trigger the immune response. Moderate immune response activation removes pathogens and avoids excessive inflammation and tissue damage. Histone demethylases (KDMs) regulate gene expression and play essential roles in numerous physiological processes by removing methyl groups from lysine residues on target proteins. Abnormal expression of KDMs is closely associated with the pathogenesis of various inflammatory diseases such as liver fibrosis, lung injury, and autoimmune diseases. Despite becoming exciting targets for diagnosing and treating these diseases, the role of these enzymes in the regulation of immune and inflammatory response is still unclear. Here, we review the underlying mechanisms through which KDMs regulate immune-related pathways and inflammatory responses. In addition, we also discuss the future applications of KDMs inhibitors in immune and inflammatory diseases.

## Facts

- Abnormal expression of KDMs play a crucial role in the pathogenesis of inflammatory diseases.
- KDMs regulate the development and function of immune cells and immune-related signaling pathways, and influence the expression of inflammatory cytokines.
- Targeting specific KDM inhibitors is potential therapeutic approach in immune and inflammation-related diseases.

## Open questions

- What are the consequences of substrates that specific inhibition of KDMs on immune cells and inflammatory diseases?
- Which KDMs in immune cells promote TLRs response?
- What are the specific protein substrates of KDMs in different immune pathways? What non-histone substrates can be targeted by KDMs?
- Which KDMs are good drug targets for the treatment of inflammatory diseases?

## Introduction

The immune response is critical in regulating the body’s immune homeostasis by protecting it from infections and injury [1–3]. Epigenetics included the dynamic regulation of different gene expression processes, such as DNA modification, chromatin remodeling, non-coding RNA regulation and histone modification. These processes were relevant to fine tuning the immune response by establishing specific gene expression patterns, through gene expression regulation at the transcriptional and post-transcriptional levels [4–6]. Histones were the first proteins identified as lysine methylation substrates, while enzymes mediating methylation and demethylation were called histone methyltransferases (KMTs) and histone demethylases (KDMs) [7, 8]. Histone methylation refered to the process of transferring methyl from methionine to histone amino acids lysine (K) or arginine (R). Demethylation of different sites could lead to different effects, including the inhibition or activation of target genes [9]. Interestingly, abnormal histone demethylation levels are closely associated with various inflammatory diseases, including kidney injury, liver injury, and autoimmune diseases [10, 11]. Increasing evidence had revealed that this modification controls cell development and regulates immune pathways [12].

In this review, we summarized the role of KDMs in immune cells’ development and the regulation of immune-related pathways, including Toll-like receptor (TLR), cGAS-STING, and IFN signal pathways. This review focuses on the role of the KDM families of proteins in immune cells and their effect on inflammation to provide a theoretical basis for developing small-molecule KDM inhibitors in treating inflammatory diseases.

## Overview of KDMs

**Fig. 1:**
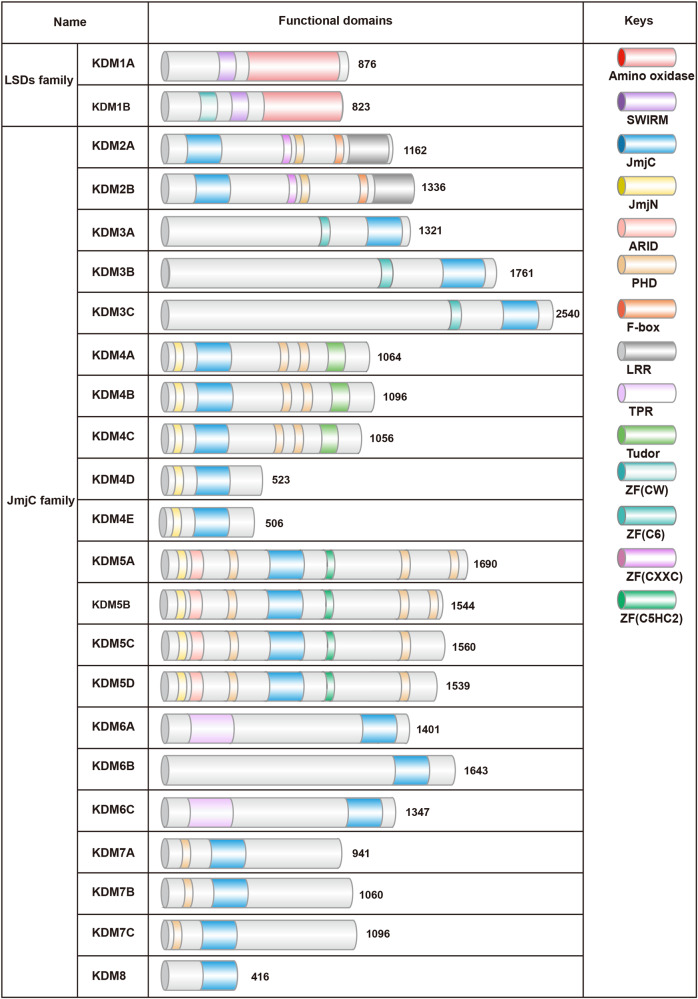
Schematic diagram of two families of human KDMs. The key domains of each KDMs are represented in colored regions, and the number of amino acids is written in each functional domain. SWIRM Swi3p, Rsc8p, and Moira domain; JmjC Jumonji C domain, JmjN Jumonji N domain, ARID AT-rich interacting domain, PHD plant homeodomain domain, F-box F-box domain, LRR Leu-rich repeat domain, TPR tetratricopeptide domain, Tudor Tudor domain, CW CW-type zinc-finger domain, C6 C6 zinc-finger domain, CXXC CXXC zinc-finger domain, C5HC2 C5HC2 zinc-finger domain.

**Fig. 2:**
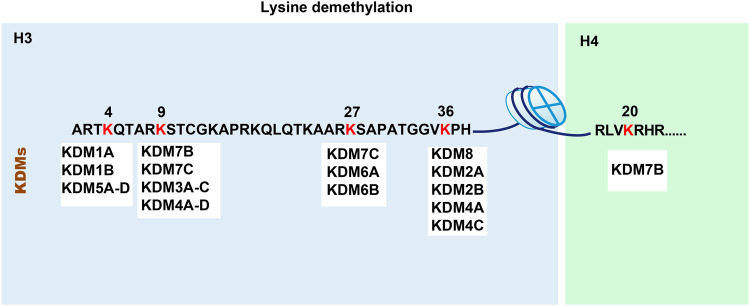
Five major demethylation sites of KDMs in histones H3 and H4 (red). The number above the site refers to the rank number of each lysine residue in the histone.

KDMs mediates the removal of methyl groups from histone lysine and arginine residues, a labile process that could regulate reversible chromatin marks [13, 14]. Histone lysine methylation had been determined as a reversible modification with the discovery of KDMs. More than 30 KDMs had been identified in humans and mice, with various motifs and essential functional domains for enzyme catalytic activity. KDMs were divided into two categories according to their catalytic mechanism (Fig. 1) :Lysine-specific demethylases (LSDs) and JmjC (Jumonji domain-containing protein) KDMs [7, 15–17]. LSDs were KDMs lacking the JmjC domain. The LSDs family were currently constituted by two members only, LSD1/KDM1A and LSD2/KDM1B [18, 19]. KDM1 had a C-terminal amine oxidase-like domain that contained the substrate and flavin adenine dinucleotide binding sites, which process the removal of monomethylated and dimethylated markers [20, 21]. KDM1 cannot remove trimethyl groups [22]. On the other hand, the KDM2-8 proteins contain a JmjC catalytic domain of ~170 amino acids with substrate specificity and demethylase activity that could remove mono-, di-, and trimethyl groups on specific sites of lysine residues [23]. This domain can remove methylation from histone H3 on lysine residues K4, K9, K27, and K36 and histone H4-K20 (Fig. 2). The JmjC domain had a similar structure to that of the Cupin protein family and contains oxygenase-catalyzed domains dependent on Fe(II) and α-KG [24]. This domain also contained a zinc finger (ZF), which brought the JmjC domain close to the C-terminus domain and was indispensable for the stability and function of KDMs. Besides the JmjC catalytic domain, all KDM proteins contain domains that interacted with the N-terminus of JmjN, providing structural integrity without catalytic function [25]. KDMs included Swi3p, Rsc8p, Moira domains, AT-rich interaction domains (ARID), plant homology domains (PHD), ZF, and Tudor domains. In addition, the F-box, Leucine Rich Repeat, and tetrapeptides domains play critical roles in their protein–protein interactions [24].

The first discovered histone demethylase containing a JmjC domain is the F-box and leucine-rich repeat protein 11 (also known as KDM2A). KDM2A had specific JmjC-dependent demethylation activity for H3K26me3 [26, 27]. Other domains of KDM2A include a ZF domain, a PHD domain, and three leucine-rich repeats. It was reported that KDM2A was recruited to CpG islands via the ZF domain, resulting in a unique chromatin state by removing the H3K36me2 modification [28]. The catalytically active ZF, ARID, and PHD domains in KDM5 separate their catalytic cores into N-terminus and C-terminus to improve substrate specificity [29, 30]. The N-terminal PHD domain binds to H3K4me, while the C-terminal PHD domain bound to H3K4me3/me2 [29]. In KDM5B, ARID was the primary DNA-binding interface through the L1 ring, which recognized the GCACA/C sequence, whereas PHD1 was involved in histone recognition and may be inhibited demethylase activity [29]. In contrast, the PHD of KDM7 had been reported to be non-essential for the catalytic activity, but was essential for substrates specificity [31]. For instance, the JmjC and PHD domains of KDM7B bind to H3K4me3, enhancing the demethylation rate on its homologous substrate, H3K4me2 [31, 32]. In addition, KDMs contain the Tudor domain, consisting of tubular structures with antiparallel beta chains present in many chromatin-associated with proteins [33]. KDM4A-C contained a conserved dual Tudor domain that determined the different binding preferences of these enzymes [34]. On the contrary, KDM4D and KDM4E do not contained a Tudor domain and used specific structures within the JmjC domain to recognize lysine [35].

## Regulation of immune cell development by KDMs

**Fig. 3:**
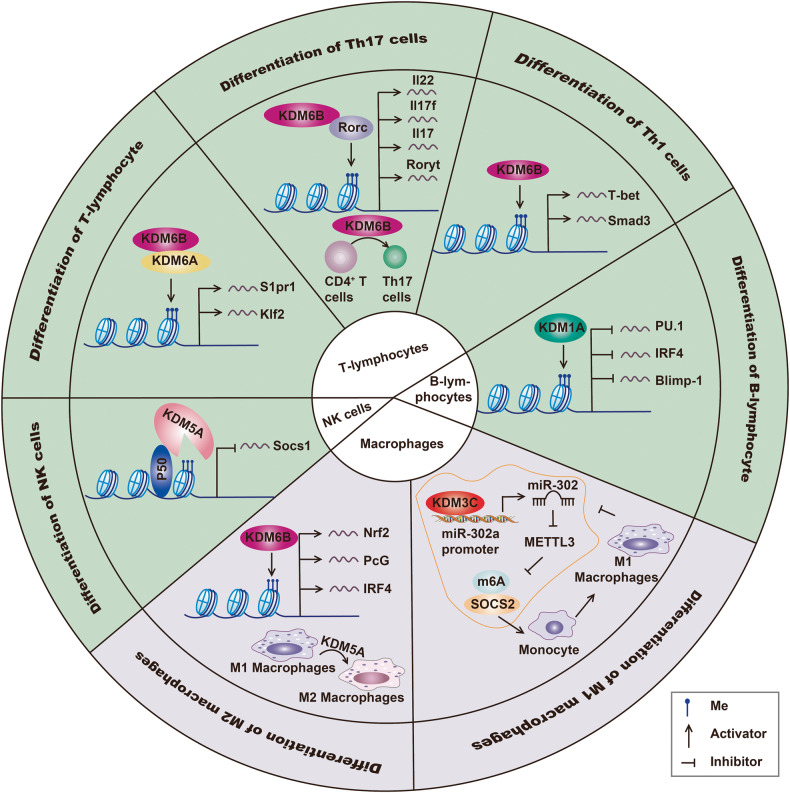
Regulatory mechanisms of KDMs in lymphocytes, NK cells, and macrophages differentiation. The KDM6 subfamily is important for the development of T lymphocytes. KDM6A and KDM6B promote the expression of S1pr1 and Klf2, crucial for the maturation of T cells, by targeting promoter H3K27me3. Subsequently, KDM6B modifies the expression of Th17-related genes including Il22, Il17f, Il17, Rorγt, and Th1-related genes including T-bet and Smad3, promoting the differentiation into Th17 and Th1 cells, respectively. During the differentiation of B cells, KDM1A takes part in repressing PU.1, IRF4 and Blimp-1 through decreasing H3K4me3, which is crucial for the formation of plasmablasts. KDM5A recruited by p50 is responsible for the downregulation of Socs1 through erasing H3K4me3 in NK cells, inducing IFN production. In macrophages, KDM3C modulates H3K9me of the miR-302a promoter and inhibits M1 macrophage differentiation via the miR-302a/METTL3 axis. Finally, the KDM6B/IFR4 axis promotes the differentiation of M2 macrophages.

Immune cells are critical components of the immune system, producing various kinds of cytokines, protecting against pathogens and clearing “nonself” substances to maintain the homeostasis of the immune system [36–41]. The development of hemopoietic stem cells (HSCs) into different immune cells, such as T lymphocytes, NK cells, B lymphocytes, and macrophages, involve selective gene expression patterns regulated by intricate mechanisms [42]. The development of many diseases is related to disequilibrium in the quantities or functions of immune cells, where epigenetic regulation plays an important role. KDMs modulate cellular differentiation by regulating the activity of cell-specific gene enhancers [43]. In addition, KDMs played a role in the lineage commitment of immune cells after stimulation by pathogens or other signals by regulating genes expression via histone demethylation [44]. Studies have shown that KDMs were essential players in innate and adaptive immunity and participate in antivirus and antitumor immunity [45–48]. KDMs control the maturation, differentiation, and function of immune cells, thus participating in the immune response and maintaining homeostasis (Fig. 3).

### Functions of KDMs in T lymphocytes development

T lymphocytes play a crucial role in regulating the immune system [49–51]. The activation of T cells must be maintained at an appropriate level to respond against pathogens and avoid overactivation and autoimmune diseases [52]. T cells were derived from lymphoid progenitors in the bone marrow, generating TCR in the thymus and developing into CD4^+^ or CD8^+^ single-positive T cells [53]. Upon stimulation by pathogens or cytokines, mature T cells in the peripheral circulation differentiate into various helper T cells (Th cells) or cytotoxic T lymphocytes (CTLs), a process widely believed to be regulated by KDMs [54]. Indeed, KDMs were shown to be associated with T cell maturation in the thymus. Histone demethylation promoted T cell maturation by suppressing marker genes of T cell precursors and inducing T cell-specific gene expression [48]. Knockdown of KDM1A inhibited the downregulation of Ctla4 and Prdm1, explicitly expressed in double negative T cells (CD4^−^CD8^−^), blocking the formation of double positive (CD4^+^CD8^+^) or CD4^+^ T cells [55]. It was also reported that KDM6A and KDM6B could induce the expression of S1pr1, a critical protein for T cell maturation, and the transcription factor Klf2 via H3K27me3 demethylation, inducing intrathymic T cell precursors to differentiate into terminal T cells [56].

Furthermore, KDMs are involved in the differentiation of CD4^+^ T cells into various subsets of Th cells that produce different cytokines [57, 58]. Wei et al. [59]. discovered that KDM1/5/6 targeted H3K4me3 and H3K27me3 and were associated with early T cell and Th cell differentiation by inducing the expression of signature genes in Th cells, such as Ifng, Il4, Il17, and Tbx21. Suzuki et al. [60]. reported that KDM1A increased the expression of transcription factors, including Tbx21, Eomes, and Runx2, which promote the development of Th1 cells and the production of IFN-γ. Similarly, Liu et al. [61]. indicated that the knockdown of KDM1A could inhibit the proliferation of CD4^+^ T cells, and decrease the secretion of IFN-γ and IL-17. Meanwhile, KDM5C was found to be downregulated in CD4^+^ T cells from multiple sclerosis (MS) patients, facilitating Th17-mediated IFN-γ secretion [62]. In addition, by interacting with lncRNA A112010, Yang et al. [63]. found that KDM5A enhanced the production of pro-inflammatory Th1 and Th17 cells by decreasing H3K4me3 and suppressing the transcription of IL-10. Furthermore, Ptaschinski et al. [64]. have shown that upon infection with the respiratory syncytial virus, ablation of KDM5B might enhance the production of IFN-β, IL-6 and TNF-α by increasing H3K4me3 in their promoters. Besides, the expression of Th2 cytokines was downregulated, resulting in chronic infection. Similarly, in the widely used autoimmune encephalomyelitis, MS mouse model, knockout of KDM6A limited the development of the autoimmune disease by suppressing CD44 expression on CD4^+^ T cells via H3K27me3 targeting Th2 cytokines expression and Th1 response blockade [65]. Using a colitis model, Qing et al. [57]. revealed that KDM6B could facilitate H3K27me3 demethylation on Th1 transcription factors, including Foxp3, Ifng and Cd44, while its deletion inhibited the transition of intestinal CD4^+^ T cells into Th1 cells. Furthermore, Liu et al. [66]. discovered that KDM6B was rapidly induced upon stimulation of TCR signaling. Overexpression of KDM6B decreased H3K27me3 in the Rorγt promoter, a crucial Th17 transcription factor, promoted Th17 differentiation, and induced the expression of cytokines such as Il17, Il17f, and Il22. However, depletion of KDM6B or treatment with GSK-J4, a KDM6A/6B specific inhibitor, reduced Rorγt expression, suppressing the generation of Th17 cells [66, 67]. Finally, KDM6B inhibited TGF-β-induced FOXP3 expression and the development of Treg cells and facilitated differentiation into pro-inflammatory phenotypes, including Th17 or Th1, thus aggravating inflammatory responses [68].

Besides CD4^+^ T cells, KDMs regulated the development of CD8+ and other subsets of T cells. After viral infection, KDM6B was rapidly upregulated in CD8 + T cells, decreasing H3K27me3 markers and enhancing CTL-mediated immune response and the formation of memory T cells [69, 70]. Similarly, depletion of KDM6A in mice treated with *Listeria monocytogenes* expressing OVA enhanced immune response after secondary infection by reducing CTLs formation through H3K27me3 regulation on Prdm1, which encoded Blimp1 and played essential roles in the development of CD8+ memory T cells [71]. In addition, KDM6B was shown to enhance the production of mucosal-associated invariant T cells (MAIT), a special type of T cells that produced Th1-like effectors. Indeed, the KDM6B co-factor α-KG was shown to enhance the effects of MAIT [72]. Intestinal intraepithelial lymphocytes (IELs) were another type of T -cells located in the intestinal epithelium. Knockout of KDM6B reduced the number of TCRαβ + CD8αα + IELs in mice, and impaired the lytic function of IELs through H3K27 demethylation on Bcl2 and FasL [44].

### The role of KDMs in the development and function of B lymphocytes

B lymphocytes were essential effectors in humoral immunity, and epigenetic regulation is required for the proliferation and differentiation of B cells [73–76]. Upon activation by thymus-dependent antigen, naïve B cells (nB) transform into plasmablasts (PB) to form germinal centers (GC), from which antibody-secreting plasma cells and memory B cells were developed. Haines et al. [77]. found that the knockdown of KDM1A in mice treated with LPS led to a shift in H3K4me1 levels in cell cycle genes and transcription factors, including PU.1, IRF3 and Blimp-1, thus suppressing the differentiation of nB into PB.

The proliferation and differentiation of B cells in GC require the assistance of Th cells, mainly T follicular helper (Tfh) cells that play indispensable roles. Hung et al. [78]. indicated that Tfh-derived signaling upregulated KDM4A and KDM4C expression, blocking normal cell cycle and inhibiting the proliferation of B cells by interacting with WDR5. Besides, depletion of KDM6A in T cells was shown to inhibit the expression of interleukin-6 receptor-α, Icos, and other genes related to the development of Tfh cells [79]. Consequently, the decreased in Tfh cell population impaired ability of B cells to produce specific IgG antibodies in response to lymphocytic choriomeningitis virus (LCMV) infection. Kei et al. [80]. highlighted the role of KDM6A in GC B cell maturation, showing that IL-4 stimulation activates the STAT6 pathway, which induces KDM6A recruitment into Bcl6 enhancer, resulting in H3K27me3 demethylation and Bcl6 induction, thereby facilitating the formation of mature B cells in GC. Interestingly, the knockout of KDM1A restrained the Bcl6-derived proliferation of GC B cells. Interestingly, BCL6 could then form a suppressive complex with KDM1A and control the regulation of Bcl6-targeted genes. Similarly, KDM6B was also associated with B cell development [81], whereby KDM6B transcription was reported to be upregulated in GC B cells compared to undifferentiated B cells [82].

### The role of KDMs in the polarization and function of macrophages

Macrophages are key components of innate immunity that regulate tissue homeostasis and participate in tissue repair by phagocytosis and clearing the debris of dead cells [83–86]. Macrophages could be polarized into functionally distinct phenotypes. Classically activated (M1) macrophages have pro-inflammatory effects, while alternatively activated (M2) macrophages manifest anti-inflammatory functions and promote tissue repair [87–90]. KDMs were involved in macrophage polarization. Indeed, KDM3C was shown to mediate M1 polarization and suppress glioma by stimulating the miR-302a/METTL3/SOCS2 axis [91]. KDM3C increased the expression of miR-302a by H3K9me1 demethylation in its promoter region, miR-302a targeted METTL3, which in turn inhibits SOCS2 via an m6A modification. While KDM6B did not influence M1 polarization, it was essential for M2 macrophage differentiation. Investigations on anti-helminth immunity revealed that KDM6B was a positive regulator of M2 activation by upregulating the transcription factor interferon regulatory factor 4 (IRF4) via H3K27me3 demethylation [92, 93]. Besides, the supplement of alpha-ketoglutaric acid (α-KG), in inflammatory diseases stimulated by the granulocyte-macrophage colony-stimulating factor, facilitated IL-4 induced M2 polarization via the KDM6B/IRF4 axis, while IFN-β reversed macrophage activation [94, 95]. In addition, stimulation of macrophages with LPS or cytokines activated of the NF-κB signaling pathway and enhanced the expression of KDM6B. The latter combined with PcG-target genes to modify downstream gene expression via H3K27me3 demethylation, fortifying macrophages’ response against inflammatory stimulation [96]. Interestingly, Huang et al. [97]. demonstrated that LPS induced the upregulation of KDM6B in bone marrow-derived macrophages, leading to decreased H3K27me3 on Nrf2 promoter, improving NLRP3 inflammasome activation in macrophages. Indeed, KDM6B-specific inhibitor GKS-J4 restrained this process and inhibited the development of colitis in sodium dextran sulfate-treated mice. Similarly, Zhuo et al. [98]. showed that the expression of KDM1A was induced, and the NLRP3 inflammasome was activated in RAW264.7 macrophages treated with pro-inflammatory Ox-LDL. Conversely, inhibition of KDM1A increased SESN2 and activated the PI3K/Akt/mTOR pathway, ultimately decreasing inflammation. In *Leishmania donovani*-infected macrophages, activated HIF-1α was shown to regulate macrophages polarization by inducing the expression of KDM5B and KDM6B, modifying H3K4 and K3K27 trimethylation and consequently suppressing M1 cytokines (TNF-α and IL-12) and promoting M2 factors such as Arg-1 [99].

### The role of KDMs in the development and function of NK cells

Natural killer (NK) cells are cytotoxic lymphocytes that rapidly fight against pathogens to maintain homeostasis [100–103]. NK cells play important roles during early viral infection by releasing granules containing perforin and granzymes and secreting critical antiviral cytokines such as interferon-γ (IFN-γ) [104–106]. Bailey et al. [107]. showed that KDM1A regulated the production of reactive oxygen species and glutathione in NK cells, while SP-2577, an inhibitor of KDM1A, weakened the cytotoxic function of NK cells by impairing oxidative phosphorylation and glycolysis. In addition, Zhao et al. [108]. observed an upregulation of KDM5A in NK cells infected with *monocytogenes* (Lm) via p50 recruitment, leading to Socs1 transcription suppression by H3K4me3 demethylation on its promoter regions. Meanwhile KDM5A activated the JAK2-STAT4 signaling pathway to induce the secretion of IFN-γ. Studies by Adam et al. [109]. indicated that KDM6B from peripheral blood or tissues of rheumatoid arthritis (RA) patients was upregulated compared to healthy controls. Furthermore, GSK-J4, an inhibitor of the H3K27me3 demethylase KDM6B, inhibited the cytokine-stimulated production of IFN-γ, TNF-α, GM-CSF, and IL-10, thus suppressing the inflammatory response in NK cells.

The invariant natural killer T (iNKT) cells are the most well-studied type of NKT cells. They had the unique ability to recognize lipid antigens presented by CD1d on the surface of antigen-presenting cells [110, 111]. Interestingly, KDM6A was enriched in the promoter of PLZF in double-positive (CD4^+^CD8^+^) T cells, while knockout of KDM6A enhanced the repressive H3K27me3 and inhibited the transcription of PLZF, which controls the production of specific TCR of lipid antigens on NKT cells [112]. Similarly, Beyaz et al. [113]. showed that KDM6A inhibited the differentiation of glycosphingolipid α-galactosylceramide (α-GalCer)-induced mouse iNKT cells by decreasing H3K27me3 in the promoters of iNKT signature genes such as Tbx21, Il2rb and Klrd1. KDM6A has been also shown to interact with JunB to establish lineage commitment of iNKT cells. Finally, studies by Northrup et al. [114]. demonstrated that Kdm6a/Kdm6b depletion downregulated cell cycle-related genes in NKT cells, alleviating hepatic injury in ConA-induced mice liver injury model.

## KDMs in the immune response and related signaling pathways

**Fig. 4:**
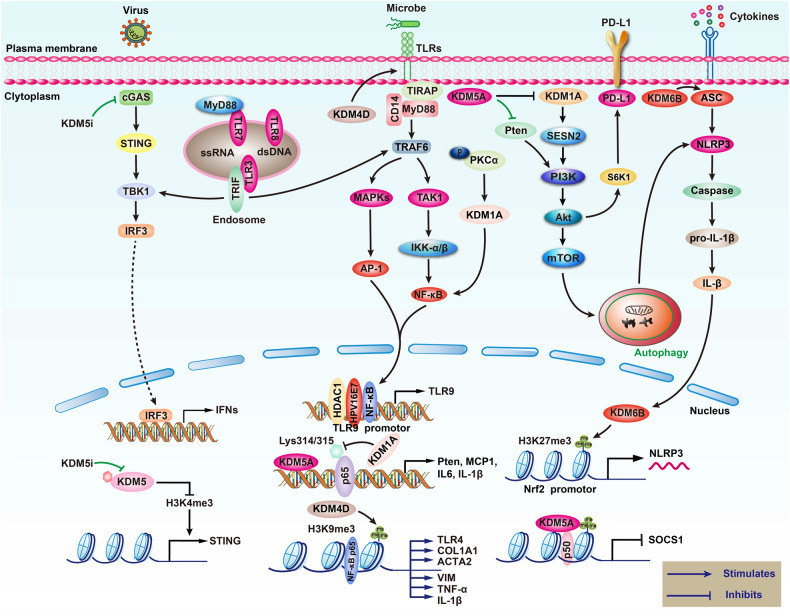
Regulation of innate immune pathways by KDMs. KDMs regulate TLR, cGAS-STING, and IFN signaling pathways to modulate inflammation and antiviral responses. (1) TLRs signaling pathway. KDM4D promotes TLR4 transcription by demethylating H3K9, thereby activating the TLR4/TIRAP/MyD88/NF-κB signaling pathway, which in turn promotes the expression of hepatic fibrosis factors COL1A1, ACTA2, VIM, and inflammatory cytokines TNF-α and IL-1β. Increasing the expression of KDM5A can inhibit Pten, activate PI3K/AKT/S6K1/PD-L1 and TLR7/8 signaling pathways, and promote the expression of cytokines and chemokines. (2) After viral infection of cells, KDM5B/5 C can inhibit STING expression by demethylation of H3K4me3, while KDM5 inhibitors increase IFN levels and improve antiviral immune response by enhancing the cGAS-STING-TBK1-IRF3 pathway. (3) Inhibition of KDM1A in cells induces the level of SESN2, which activates the autophagy-related PI3K/Akt/mTOR pathway, ultimately inhibiting the activation of the NLRP3 inflammasome. At the same time, it increases KDM6B and promotes ASC/NLRP3/Caspase, hence promoting the inflammatory response.

Immune responses depend on how immune cells react to stimuli. Pathogens or inflammatory signals recognized by pattern recognition receptors (PRRs) or cytokine receptors are transduced along various signaling pathways, activating nuclear transcription factors and inducing the production of effector molecules to stimulate the immune response. KDMs are involved in the regulation of gene transcription and thereby participate in the control of immunity (Fig. 4).

### KDMs regulate the recognition and transduction of stimulatory signals

PRRs are important receptors on innate immune cells, which include TLRs, RNA helicases retinoic acid-inducible gene I that recognizes cellular RNA, and the cGAS-STING pathway recognizing DNA [115, 116]. Among these, TLR9 was shown to bind to the CpG motif of viral double-stranded DNA and plays critical roles in anti-viral immunity. Hasan et al. [117]. found that KDM5B was recruited by the E7 protein of HPV16 via NF-κB signaling pathway in infected human epithelial cells. KDM5B interacted with HDAC1 to downregulate TLR9 expression by modulating its promoter activity, thus suppressing viral infection. As for the regulation of the cGAS-STING pathway, Wu et al. [118]. reported that KDM5B/5C suppressed the expression of STING after viral infection by removing H3K4me3 modifications. An inhibitor of KDM5 activated the cGAS-STING-TBK1-IRF3 axis to boost IFN production and anti-viral immune response. Wang et al. [119]. indicated that KDM5A activated the TLR7/8 and PI3K-AKT-S6K1 pathways to promote PD-L1 expression. CCl4-induced liver fibrosis studies by Dong et al. [120]. showed that upregulation of KDM4D catalyzed H3K9 demethylation of TLR4 promoters, which in turn activated NF-κB signaling pathway to induce fibrosis. Taken together, these studies support that KDMs regulated stimuli recognition by PRRs.

The transduction of PRR signals relies on different cellular pathways, and KDMs play two roles in this process [121]. First, KDMs influence the activation of these pathways. For instance, in NK cells stimulated with Lm infection or IL12/18, KDM5A was recruited to the promoters of SOCS1 and inhibited its expression via H3K4me3 demethylation, which activated STAT4 signaling and led to IFN-γ production [108]. Similarly, TFN-α-induced endothelial inflammation was shown to be dependent on the activation of Jagged-1 and the Notch activator ADAM17 and by KDM6A/6B-induced H3K27me3 demethylation [122]. In addition, KDM7B inhibited IFN-γ-target genes by removing H4K20me1 and blocking the transduction of IFN-γ signals [123]. Second, KDMs regulated several transcription factors, and NF-κB was one of the most critical effectors mediating TLR or cytokine-target genes. Kim et al. [124]. indicated that in sepsis combined with lung injury, activated PKCα was transported into the nucleus and induced the interaction of KDM1A with p65, which led to p65 demethylation, suppressing inflammatory response via PKCα-KDM1A-NF-κB axis and ultimately reducing the death rate in mice. LPS could also induce the expression of FBX11 (KDM2A) via NF-κB pathway. However, KDM2A targeted the lysine K218 or K221 of p65 and inhibited p65 expression through demethylation on those sites, constituting negative feedback [125]. Through association with deubiquitinase USP38, KDM5B antagonized NF-κB signaling and inhibited LPS-stimulated activation of Il6 and Il23a promoters [126]. Connor et al. [127]. demonstrated that KDM6B was upregulated in epithelial cells infected with S. *pneumoniae*. Moreover, KDM6B removed H3K27me2 and recruited p65 to the Il11 promtoer, regulating the production of IL-11 and other cytokines including IL-1β and TNF-α. Studies of Higashijima et al. [128]. showed that KDM6A and KDM7A synergistically occupied the NF-κB binding site in TNF-α-induced endothelial cells, enhancing the expression of p65 target genes through the regulation of H3K9me2 and H3K27me3. Similarly, KDM7C was recruited by p65 to the promoters of Tnf, Ccl4 and Il11 upon LPS stimulation, which activated their expression by removing H4K30me3 [129]. Finally, KDM4A was also recruited by p65 after viral infection, inducing the production of INF-β through H3K9me3 demethylation [130].

### KDMs mediate the expression and function of effector molecules

KDMs positively regulate inflammatory factors [131]. During the process of innate immunity, KDM6A/6B removed the repressive H3K27me3 and promoted IL-6 mediated inflammation [132–134]. Indeed, the KDM6 inhibitor GSK-J4 was shown to suppress TNF-α production upon LPS stimulation possibly via the regulation of TNFA transcription by KDM6A/6B [135]. During the development of dendritic cells (DCs), GSK-J4 significantly decreased the expression of the co-stimulatory molecules CD80/CD86, upregulated anti-inflammatory TGF-β1 and down-regulated pro-inflammatory IL-6, thus suppressing the activation of DCs [134, 136]. GSK-J4 also promoted IFN-γ secretion, since KDM6B get recruited by Cbx2 to IFN promoter to enhance its transcription through H3K27me3 demethylation [109, 137]. KDM6A was also shown to interact with the methyltransferase MLL4 and regulated the enhancers of Ifnb, thus promoting IFN-β expression [133]. In addition, KDM2B could interact with Brg1, which was the core component of the SWI/SNF complex, increasing chromatin accessibility of the Il6 promoter and inducing its expression in macrophages and DCs [131].

Conversely, KDM1 and KDM5 proteins that targeted the promoting H3K4 methylation often disrupt chromatin accessibility and inhibited gene expression [138, 139]. KDM1A could directly inhibit IL-1β, IL-6, IL-8 and the classic complement components, working synchronously with HDAC1 to restrain inflammation [140]. The endotoxic shock was shown to cause KDM1A suppression, which protected against inflammatory response over-activation, leading to excessive proliferation of myeloid progenitors, producing IL-1β and TNF-α and forming cytokine storms [138]. Furthermore, KDM5C could interact with Polycomb group factor 6 to regulate the H3K4me3 of Il11b and Ciita, suppressing the activation of DC and T cells [139]. By stimulating mouse embryonic fibroblasts with poly(I:C), Yu et al. [141]. showed that KDM5A was recruited to the IFN-β promoter, inhibiting its transcription via H3K4me3 demethylation.

## KDMs and inflammatory diseases

There was growing evidence that abnormal expression of KDMs was associated with a variety of inflammatory diseases, including mastitis, liver fibrosis, inflammatory kidney injury, inflammatory pulmonary injury, colitis, autoimmune diseases, and inflammation-related cancers [96, 142–144]. The expression of KDMs was up-regulated or down-regulated in these diseases, and the transcription and secretion of inflammatory mediators were regulated through specific demethylation or activation of inflammation-related pathways, thus promoting the occurrence and development of inflammatory diseases [133, 145–148] (Table 1). Since there are so many inflammatory diseases that may involve the dysfunction of KDMs, this review we discuss six of the following inflammatory diseases.

**Table 1:** Summary of functions and mechanisms of KDMs in inflammatory diseases and respective targeted therapy.

	Inflammatory type	Mechanisms or pathways	Function	Targeted treatment for the KDM family	Reference
KDM1A	Mastitis	NF-κB pathway	Promotes the NF-κB signaling pathway and the secretion of inflammatory factors TNF-α, IL-6, and IL-8	GSK-LSD1	[153]
	Nephritis	TLR4/NF-κB/JNK pathway	Binding to the TLR4 promoter promotes inflammatory cytokine production through the downstream NF-κB/JNK signaling axis	shLSD1	[175]
	Rheumatoid arthritis, RA	CD4 + T cell	Promotes CD4 + T cell proliferation and the production of the inflammatory cytokines IL-17 and IFN-γ	si-LSD1, sh-LSD1	[61]
KDM3A	Hepatic fibrosis	Peroxisome proliferator-activated receptor γ (PPARγ)	The KDM3A expression was downregulated during HSC activation, and the reduced binding to the PPARγ promoter promoted the expression of liver fibrosis markers and the disappearance of lipid droplets	si-JMJD1A, sh-JMJD1A	[165]
KDM4B	Rheumatoid arthritis, RA	STAT3 pathway	Promotes the secretion of various proinflammatory cytokines produced by fibroblast-like synovial cells (FLS) by activating the STAT3 signaling pathway	shKDM4B	[193]
KDM4D	Hepatic fibrosis	NF-κB pathway	Overexpression of KDM4D promotes liver fibrosis through the upregulation of TLR4/NF-κB	si-KDM4D, AAV-sh-KDM4D	[120]
	Inflammatory bowel disease, IBD	Hedgehog signal	The inflammatory factor TNF-α induces upregulation of KDM4D and activates Hedgehog signaling to promote cell proliferation, leading to inflammatory-related cancers	5-c-8HQ	[186]
KDM5A	Renal fibrosis	NF-κB pathway	Recruitment of KDM5A to NF-κB signaling attenuated renal sepsis-induced failure	CPI-455, KDM5A-IN-1	[174]
KDM6B	Mastitis	TLR4/NF-κB pathway	Upregulated KDM6B binding to the TLR4 promoter region promotes TLR4 and downstream NF-κB signaling	GSK-J1	[152]
	Hepatic fibrosis	TGF-β/SMAD pathway	Upregulated KDM6B inhibits TGF-β/SMAD signaling and reduces the transformation of HSCs; Upregulated cell cycle regulators such as p21 and Gadd45 promote cell senescence, and inhibit the expression of extracellular matrix (ECM) and α-smooth muscle actin (α-SMA)	GSK-J4	[162]
	Renal fibrosis	TGF-β1/Smad3 pathway; Notch pathway	Upregulation of KDM6B expression exerts anti-fibrotic effects through the inhibition of both the TGF-β1/Smad3 pathway and the Notch signaling pathway	GSK-J4, si-JMJD3	[170]
	Injury of lungs	Akt/JNK; TGF-β/Smad3 pathway	KDM6B promotes Akt/JNK and TGF-β/Smad3 signaling to exacerbate asthma and airway inflammation	GSK-J4	[180]
	Inflammatory bowel disease, IBD	JAK2/STAT3 pathway; NF-κB pathway	KDM6B participates in JAK2/STAT3 pathway after activation by STAT3, which enhances the transcription of key inflammatory genes and promotes NF-κB signaling and aggravates intestinal inflammatory damage	GSK-J4	[185]
	Rheumatoid arthritis, RA	Proliferating cell nuclear antigen (PCNA)	Upregulation of KDM6B expression promotes the transcription of the PCNA gene, which mediates the migration and proliferation of fibroblast-like synoviocytes (FLS) and aggravates arthritis	GSK-J4, si-JMJD3	[190]

### Mastitis

Mastitis was a disease characterized by the inflammation of the breast parenchyma [149, 150]. In a mouse model of mastitis, LPS was shown to activate the NF-κB signaling pathway through TLRs recognition on mammary epithelial cells, promoting the production and release of inflammatory factors [151]. In a similar mouse model of mastitis induced by LPS and respective in vitro cell experiments, Wang et al. [152]. found that the expression of KDM6B in mammary epithelial cells was increased, and H3K27me3 demethylation in the promoter region induced the expression of TLR4 and activated downstream transduction of pro-inflammatory NF-κB signaling. Similarly, GSK-J1 suppressed KDM6B and therefore decreased the expression of inflammatory cytokines TNF-α, IL-1β and IL6. KDM1A was also a key pro-inflammatory regulator of mastitis, as demonstrated by Wang et al. [153] These authors had found that a KDM1A inhibitor (GSK-KDM1A) could up-regulate histone H3K4me2 and H3K9me2, which inhibited the NF-κB signaling pathway to reduce the expression of inflammatory factors including TNF-α, IL-6, and IL-8, thus reducing the inflammatory reaction in mammary epithelial cells.

### Hepatic fibrosis

The central step of hepatic fibrosis development was the transformation of hepatic stellate cells (HSCs) into activated myofibroblasts, which played an important role in the regulation of hepatic fibrosis [154–159]. Studies have shown that KDMs control the activation of HSCs [160, 161]. Yan et al. [162]. proposed that KDM6B was a pivotal negative regulator of CCl4 and BDL-induced mouse liver fibrosis. On the one hand, KDM6B inhibited TGF-β/SMAD signaling by increasing BAMBI expression, thus reducing the transformation of HSCs. On the other hand, KDM6B promoted cell senescence by upregulating p21 and Gadd45, and suppressed the expression of extracellular matrix (ECM) proteins and α -smooth muscle actin. Ming et al. [163]. found that KDM4 was down-regulated in HSCs with liver fibrosis, and the overexpression of KDM4 could synergize with SREBP2 to activate miR-29 transcription and inhibit HSCs activation. Furthermore, Li et al. [164]. observed that Brg1 recruited KDM4 and interacted with β-catenin to enhance Wnt signaling in hepatocytes, thus promoting liver regeneration after injury. In contrast, Fang et al. [120]. found that the expression of KDM4D was up-regulated during HSC activation, which further activated the TLR4/NF-κB signaling pathway and promoted liver fibrosis. In addition, Yan et al. [165]. showed that in a CCl4-induced mouse liver fibrosis model, the expression of KDM3A in HSCs was down-regulated, whereas KDM3A overexpression induced the demethylation of H3K9me2 in the PPARγ promoter, leading to inhibition of HSC activation. A recent study supported that KDM5 proteins were key factors for sex differences in alcohol-related liver disease (ALD). Indeed, KDM5B and KDM5C promoted liver fibrosis through increasing transcription of fibrosis and inflammation related genes, such as Col3a1, Itgav, and Gabarapl1 and down-regulating the expression of AhR only in female mice and female ALD patients [166].

### Inflammatory kidney injury

Kidney fibrosis was generally believed to be central to the progression from chronic kidney injury to end-stage renal disease [167], and it had been reported that the renal fibrosis signaling pathway was regulated by epigenetic mechanisms [168, 169]. Indeed, Yu et al. [170] showed that the expression of KDM6B was increased in a mouse model of renal fibrosis, inhibiting DNA methyltransferase 1 to promote Smad7 expression, and ultimately decreasing the TGF-β1/Smad3 pathway. On the other hand, KDM6B can upregulate FBXW7, a negative regulator of Notch, inhibiting the Notch signaling pathway and ultimately exerting anti-fibrotic effects. Likewise, He et al. [171] showed that KDM6B and miR-93-5p were up-regulated in mice with acute kidney injury (AKI) and that the expression of inflammatory factor TNF-α was reduced through demethylation of H3K27me3, thus alleviating kidney injury. Feng et al. [172] found that low expression of KDM6B could promote neointimal hyperplasia and inflammatory cell infiltration, which might cause vascular stenosis and access failure in renal dialysis patients. Chen et al. [173] found that KDM6A was a key pro-inflammatory factor of diabetic nephropathy (DKD) in db/db mice. KDM6A upregulation in the kidneys of diabetic mice promoted the expression of various inflammatory factors such as IL-1β, IL-8 and IL-6, leading to the deterioration of DKD. Liu et al. [174] demonstrated that dexmedetomidine (DEX) could alleviate LPS-induced AKI in mice by inhibiting the NF-κB signaling pathway and the expression of KDM5A. In addition, in HBV-infected HK-2 cells, enrichment of KDM1A reduced the level of H3K9me1/2 in the TLR4 promoter region, leading to the production of inflammatory cytokines through the TLR4-NF-κB/JNK axis and renal inflammatory response exacerbation [175].

### Inflammatory pulmonary injury

Asthma was a chronic inflammatory disease of the airways, where exposure to allergen activate immune cells and triggered inflammation and immune responses [176–178]. Bajbouj et al. [179] showed that treating lung fibroblasts with IL-13 induced the expression of KDM4B and H3K36me3 demethylation, thus promoting nuclear translocation. Meanwhile, early inhibition of KDM4B activity could postpone or even prevent airway fibrosis in asthmatic patients. In a mouse asthma model established by Yu et al. [180], a KDM6B inhibitor (GSK-J4) was shown to reduce airway inflammation, hyperreactivity and remodeling by blocking Akt/JNK and TGF-β/Smad3 signaling. In addition, it was showed that DEX could promote the expression of keratinocyte growth factor (KGF-2) by down-regulating KDM6B and ameliorating endothelial barrier dysfunction in ischemia/reperfusion related lung injury [181]. He et al. [182] found that Cu, Zn-SOD could activate STAT6, which stimulated the expression of KDM6B and interacted with KDM6B to induce profibrotic M2 gene promoters, hence stimulating macrophage polarization to the M2 phenotype and exacerbating pulmonary fibrosis. In addition, Fraszczak et al. [183] found that mice with impaired binding of GFI1 to KDM1A, displayed increased expression levels of serum IL-6, TNF-α, and IL-1β, while their alveolar macrophages secreted a large amount of cytokines that ultimately led to increased mortality.

### Colitis

A large amount of evidence suggests that histone demethylation played an important role in intestinal inflammatory responses and the transformation to colorectal cancer (CRC) [184]. Ma et al. [185] showed in necrotizing colitis mice that KDM6B was activated by STAT3 and participated in JAK2/STAT3 pathway to enhance the transcription of inflammatory genes. Besides, KDM6B might interact with NF-κB to activate the transcription of TNF-a/Il1b/Il6 and ultimately aggravated intestinal inflammatory damage. Moreover, Zhuo et al. [186] found that TNF-α induced high expression of KDM4D in colitis, which activated Hedgehog signaling to promote colon regeneration and CRC cell proliferation. Moreover, Sun et al. [187] reported that AMPK could recruit KDM1A to the Cdx2 promoters, upregulating Cdx2 expression and enhancing intestinal barrier function and epithelial differentiation. Finally, Parmar et al. [188] demonstrated that KDM1A was required to restore the intestinal barrier against pathogens invasion by promoting the maturation of intestinal epithelial goblet cells in mice with colitis induced by bacterial or parasitic infection.

### Autoimmune diseases

The role of KDMs in gene expression regulation played an important role in the occurrence and development of autoimmune diseases [189]. Rheumatoid arthritis (RA) was an aggressive joint disease resulting from immune imbalance and excessive inflammatory responses. Wan et al. [190] showed that the up-regulated expression of KDM6B in RA promoted arthritis development by inducing the transcription of PCNA, which mediated the migration and proliferation of fibroblast-like synoviocytes (FLS). Subsequent studies showed that GSK-J4, a KDM6A/6B inhibitor, suppressed the expression of RANKL and the production of TNF-α and GM-CSF in NK cells, reducing osteoclast generation and inflammatory response [109]. Wu et al. [191] found that in synovioblasts of RA patients, cystathionine-gamma-lyase inhibited the progression of joint inflammation by reducing the expression of KDM6B, suppressing the transcription of TLR2 and some inflammatory factors including TNF-α and IL-6. Meanwhile, Zhao et al. [192] showed that in collagen-induced arthritis (CIA) mice, GSK-J4 inhibited IL-6 transcription in macrophages, thus alleviating RA symptoms. Likewise, Zhang et al. [193] found that KDM4B promoted the secretion of various pro-inflammatory factors and aggravated RA by activating STAT3 signaling pathway in FLS. Heng et al. [194] clarified that in systemic lupus erythematosus, the expression of KDM6B was increased, and its H3K27me3 demethylation activity enhanced the expression of CD11a, thus promoting T cell auto-reactivity and production of autoantibodies. MS is an autoimmune disease of the central nervous system, mediated by T cells. High expression of KDM6A on the X chromosome was found in an MS mouse model, which could explain the high prevalence of this disease in women. KDM6A could also downregulate Th1 and Th2 activation pathways in mouse CD4^+^ T cells, promoting neuroinflammatory signaling via TLRs and IL-17 [65].

## KDMs inhibitors in the treatment of immune-related diseases

Many small-molecule inhibitors of KDMs have been developed in recent years with the understanding of the structures and mechanisms of KDMs [195]. According to existing research, some molecules have broad prospects for clinical use, such as the KDM1A inhibitor TCP, the KDM6-specific inhibitor GSK-J1/4 and the inhibitor of JmjC family JIB-04 (Table 1).

TCP was first approved for clinical use as a monoamine oxidase inhibitor to treat depression and was later shown to inhibit KDM1A as well [196]. Motivated by these discoveries, further studies developed dozens of inhibitors with increased specificity for KDM1A, some of which have entered clinical trials including ORY-1001 (Iadademstat), ORY-2001 (Vafidemstat), IMG-7289 (Bomedemstat), and CC-9001 [197, 198]. Studies on these drugs mainly focused on solid tumors such as small cell lung cancer and hematological diseases such as non-Hodnkin lymphoma and acute myeloid leukemia [199–203]. Despite the lack of clinical studies, KDM1A inhibitors have shown significant benefits in pre-clinical models of inflammatory diseases. KDM1A was shown to be upregulated and was associated with excessive inflammation in patients with hepatitis B virus (HBV)-associated glomerulonephritis. While in HBV transgenic mice, TCP treatment blocked the TLR4-NF-κB-JNK pathway and reduced inflammatory response [175]. ORY-2001 was shown to alleviate neuroinflammation by preventing the development of demyelination and inhibiting T cell infiltration in the spinal cord, hence could be beneficial in treating MS [204]. Importantly, ORY-2001 showed excellent safety and was proven to be able to penetrate the central nervous system [198].

GSK-J1/4, developed by Kruidenier et al. [135] in 2012, selectively inhibits KDM6 family members and is the most widely used inhibitor for JmjC demethylases. GSK-J1/4 showed anti-inflammatory effects in most in vivo and in vitro experiments, which was mainly achieved by inducing immune tolerance of NK cells and DCs. GSK-J4 was shown to suppress IFN-γ production by NK cells, thus reducing inflammatory injury of RA patients and impairing NK cell induced formation of osteoclasts and joint erosion [109]. GSK-J4 also reduced the inflammation of synovial fibroblasts and attenuated joint damage in CIA mice [191]. DCs produce cytokines and regulate the function of macrophages and Th cells to indirectly induce inflammatory response as well. However, GSK-J1/4 treatment transformed pro-inflammatory DCs into tolerogenic DCs that restrained inflammation and reduced the expression of the costimulatory molecules CD80/CD86 and pro-inflammatory IL-6, IFN-γ and TNF [134]. Additionally, tolerogenic DCs suppressed the formation of M1 macrophages and pro-inflammatory Th cells (Th1 and Th17), supporting the development of Treg cells [205]. In MS, injection of GSK-J4-treated DC cells into EAE mice reduced CD4^+^ T cell infiltration in the central nervous system and improved inflammatory responses [134]. Likewise, GSK-J4 promoted Treg differentiation and IL-10 secretion, which attenuated inflammatory responses and reduced IL-6 and IL-17 production in murine colitis [206]. Similarly, GSK-J1 treatment decreased the proportion of Th17 cells in mouse colon tissue and pro-inflammatory cytokines IL-17A, IL-22, IL-21, and transcription factors RORγt and STAT3 secreted by Th17 cells were suppressed, while Treg cell-related IL-10, TGF-β, and FOFP3 expression were increased [205]. Additionally, GSK-J4 could reduce the inflammatory response in diseases including osteoarthritis, asthma, and mastitis in animal models [152, 180, 207]. The clinical value of other JmjC family inhibitors such as the KDM4/5 family inhibitor JIB-04 and the KDM4C inhibitor SD70 warrants further exploration [208, 209].

## Conclusions and perspectives

In complex and elaborate immune responses, epigenetic modulation of KDMs is an critical for maintaining inflammatory response progression. Different KDM family proteins fine-tune the switch of gene expression by manipulating activatory or inhibitory histone methylation markers, thus participating in various links of immune cells and inflammatory activity. During the initial phase of the immune response, KDMs regulate the activation of innate immune cells, such as macrophages, DCs and NK cells, and activate inflammation by regulating PRR and downstream pathways, or directly regulating the transcription of inflammatory factors at gene promoters. Furthermore, KDMs can induce the differentiation and function of adaptive immune cells, such as T and B cells by altering intracellular gene expression patterns. Finally, during the resolution phase, KDMs can regulate immune tolerance by macrophages, DCs, and Treg cells to prevent immune overreaction.

Dysfunction of KDMs can lead to insufficient or excessive immune response, which is associated with the development of various inflammatory diseases, autoimmune diseases, and tumors. Remarkably, the regulation of KDM6A/6B is the most extensively studied. Therefore, small molecule inhibitors targeting these KDMs may provide new strategies for the treatment of inflammatory diseases. However, there are still some obstacles to their clinical application. First, the mechanism of action of KDMs in different diseases remains to be further clarified. Second, there are no clinical trials on related diseases, and the safety and efficacy of GSK-J1/4 widely used in animal experiments remain to be discussed. Finally, the interaction between histone demethylation and other epigenetic regulatory systems is not clear, and only a clearer understanding of these mechanisms can help to avoid adverse drug reactions. In conclusion, we believe that the role of KDMs in inflammation and immune response and their clinical prospect warrant further investigations, and we expect that KDMs will improve the understanding and treatment of related diseases in the future.
